# Factors Predicting Physical Activity Among Children With Special Needs

**DOI:** 10.5888/pcd10.120283

**Published:** 2013-07-18

**Authors:** Shahram Yazdani, Chu Tang Yee, Paul J. Chung

**Affiliations:** **Author Affiliations:** Chu Tang Yee, University of California, Los Angeles, California; Paul J. Chung, David Geffen School of Medicine at the University of California, Los Angeles (UCLA), and Mattel Children’s Hospital UCLA, UCLA Fielding School of Public Health, RAND Corporation, Los Angeles, California. Dr Yazdani is affiliated with both the David Geffen School of Medicine at UCLA and Mattel Children’s Hospital UCLA.

## Abstract

**Introduction:**

Obesity is especially prevalent among children with special needs. Both lack of physical activity and unhealthful eating are major contributing factors. The objective of our study was to investigate barriers to physical activity among these children.

**Methods:**

We surveyed parents of the 171 children attending Vista Del Mar School in Los Angeles, a nonprofit school serving a socioeconomically diverse group of children with special needs from kindergarten through 12th grade. Parents were asked about their child’s and their own physical activity habits, barriers to their child’s exercise, and demographics. The response rate was 67%. Multivariate logistic regression was used to examine predictors of children being physically active at least 3 hours per week.

**Results:**

Parents reported that 45% of the children were diagnosed with attention deficit hyperactivity disorder, 38% with autism, and 34% with learning disabilities; 47% of children and 56% of parents were physically active less than 3 hours per week. The top barriers to physical activity were reported as child’s lack of interest (43%), lack of developmentally appropriate programs (33%), too many behavioral problems (32%), and parents’ lack of time (29%). However, child’s lack of interest was the only parent-reported barrier independently associated with children’s physical activity. Meanwhile, children whose parents were physically active at least 3 hours per week were 4.2 times as likely to be physically active as children whose parents were less physically active (*P* = .01).

**Conclusion:**

In this group of students with special needs, children’s physical activity was strongly associated with parental physical activity; parent-reported barriers may have had less direct effect. Further studies should examine the importance of parental physical activity among children with special needs.

## Introduction

Recent evidence suggests that although childhood obesity has reached epidemic proportions in the United States, this problem appears to be even more prevalent among children with developmental or learning disabilities (1–4). Obesity poses medical problems such as sleep apnea, insulin resistance, and hyperlipidemia, as well as psychosocial consequences such as social isolation, pain, and depression (5,6). Moreover, studies suggest that adolescent obesity may persist into adulthood and be a predictor of additional comorbidities later in life (7). Thus, obesity can significantly worsen the already fragile health of children with special needs.

Youth with disabilities may require unusual diets, have limited nutritional options, and use medications such as corticosteroids, which may predispose them to obesity; a large proportion, meanwhile, may lack adequate physical activity (3,8–10). Liou et al suggested that nearly 75% of people with disabilities do not participate in enough physical activity to achieve physical health benefits (5). Physical activity can also provide opportunities to interact in appropriate social settings and has been correlated with improvement in cognitive function, attention, and psychological health in children (11–15). Thus, physical activity may be a pivotal factor not only in preventing obesity and health risks associated with weight gain but also in promoting healthy cognitive, psychosocial, and physical development in children with special needs.

Numerous factors have been proposed to explain why children with developmental or learning disabilities participate in less physical activity and have higher obesity prevalence than other children. Chen et al postulated that the energy and effort invested by parents in managing their child’s chronic illness may prevent them from dedicating time to physical activity; others have mentioned parent lack of motivation and child physical limitations as possible causes of inadequate physical activity in these children (3,4,16). In a series of town hall meetings with parents and community advocates aimed at improving nutrition and physical activity among children, parents identified factors such as lack of access to safe and appropriate places for physical activity, time constraints, and lack of parental awareness and motivation as major barriers to getting adequate physical activity (17). Furthermore, parental attitudes and perceptions about physical activity have been identified as key determinants of physical activity in childhood (16,17). The effect of parental behavior on physical activity in children with special needs, however, has yet to be investigated.

To our knowledge, no studies have carefully examined child and parent barriers to physical activity among children with special needs, particularly children with developmental or learning disabilities. The objective of this study was to identify barriers to physical activity among children with special needs by using a cross-sectional survey of parents.

## Methods

From October 2010 through April 2011, in a collaborative effort with the teachers and administrative staff at Vista Del Mar School in Los Angeles, we created and distributed a set of questionnaires to the parents, legal guardians, or other primary caregivers of the children attending this school (n = 171). Vista Del Mar is a nonprofit school serving children from kindergarten to 12th grade, dedicated to the educational and therapeutic needs of children with chronic conditions and disabilities, primarily serious mental health problems, developmental difficulties, and emotional or behavioral issues. Questionnaires (and descriptions of the study for informed consent) were distributed by the teachers and administrative staff, and one respondent per family was asked to anonymously return surveys in the provided unmarked pre-stamped envelopes. The overall response rate was 67%. This study was reviewed and approved by the University of California Los Angeles (UCLA) Institutional Review Board.

We created a conceptual framework for our questionnaire that was mostly based on our own observation and the previously published literature (3,4,16–18). Children with special needs may face barriers to physical activity greater than those of typical children. Some barriers may be specific to the physical or emotional nature of their special needs. Others, however, may be related to parent and family factors that are exacerbated by their special needs (eg, parent time or energy devoted to their special needs may limit time or energy for child physical activity). Still others may be related to the inability of local environments and resources to successfully accommodate or anticipate their special needs.

The product was an assessment tool composed of 25 questions, designed to measure caregivers’ self-reported assessment of children’s physical activity level and possible barriers to physical activity. The survey included demographic questions dealing with such issues as family structure, socioeconomic status, child’s disability, and the health services received. The remaining questions dealt with the amount and adequacy of children’s physical activity, caregivers’ own time commitment and attitude toward physical activity, and perceived barriers to children’s participation in physical activity. To improve the validity and reliability of our survey, we piloted our questionnaire with 30 parents of children with special needs at a health fair specifically organized for these children and debriefed them to obtain their feedback regarding the format, clarity, and types of questions. No formal validity and reliability testing was conducted.

Our primary outcome was how much moderate-to-vigorous physical activity their child participated in each week. We asked, “How much does your child exercise (eg, organized sports, running/brisk walking, swimming)?” We did not ask about the location of the activity or explicitly define moderate-to-vigorous physical activity. Answer options were none, 1 to 2 hours per week, 3 to 5 hours per week, and 6 or more hours per week.

### Statistical analysis

We initially used bivariate logistic regressions to examine potential predictors of children getting 3 or more hours per week of physical activity. Although the Centers for Disease Control and Prevention (CDC) recommends at least 1 hour per day of physical activity for children, there are no current guidelines for children with serious functional disabilities. Therefore, we empirically selected 3 or more hours per week on the basis of answer distributions in our population and sensitivity analyses using other cutoffs. Any predictors that were associated with child physical activity within the commonly used cutoff of *P* < .20 were included in the multivariate regression. All analyses were conducted using Stata version 11 software (StataCorp LP, College Station, Texas).

## Results

A total of 114 parents and other primary caregivers (114/171 = 67% response rate) participated in this survey (Table 1). The majority of respondents were female (82%) and had a mean age of 44 years; the average age of the children was 12 years (range, 4−21 years). Most of the children were male and had reportedly been diagnosed by their physician as having 1 cognitive or learning disability or a combination of such disabilities. Only a small proportion had been diagnosed with some form of physical limitation (Table 1).

**Table 1:** Demographics of Children and Parents and Physical Activity Habits

**Survey Question**	**% (n = 114)**
**Relationship to child**	
Biological parent	77
Adoptive/foster parent	7
Legal parent	6
Not specified	9
**Sex of respondent**	
Female	82
Male	12
**Race/ethnicity**	
White	35
Latino	26
African American	30
Other	9
**Level of education**	
Less than high school graduate	16
High school graduate	12
Some college, no bachelor’s degree	36
Bachelor’s degree	17
Graduate degree	19
**Sex of child**	
Male	77
Female	23
Child’s diagnosis	
Physical limitations	8
Attention deficit hyperactivity disorder (ADHD)	45
Autism	38
Learning disabilities	34
Family health status	
Respondent reported in excellent health	18
Child reported in excellent health	26
**Type of household**	
Two parents	51
One parent	42
Other	7
**Household income, $**	
<25,000	35
25,000-49,999	26
≥50,000	39
**Level of physical activity**	
Children are physically active ≥3 h/wk	53
Parents are physically active ≥3 h/wk	44
Children are physically active <3 h/wk	47
Parents are physically active <3 h/wk	56
Parents felt their child needed more exercise	71
Doctor had recommended child to exercise more	41
**Parents’ preferred type of exercise for their child**	
With similarly disabled children	18
With variously disabled children	5
With nondisabled children	19
No preference	65

Our survey showed that 47% of children and 56% of parents reported being physically active less than 3 hours per week. Seventy-one percent of the parents felt that their child needed to be more physically active, and 41% reported that their child’s doctor had also recommended more physical activity. Most parents (65%) indicated that they had no preference as to whether their child was physically active with other children who shared similar disabilities, had other disabilities, or had no disabilities.

The most common barriers to child physical activity cited by parents were child’s lack of interest (43%), lack of programs appropriate for children with special needs (33%), too many behavioral problems (32%), and parent’s lack of time (29%) (Table 2). In multivariate regressions, however, only child’s lack of interest was associated with parent-reported hours of child physical activity (odds ratio [OR] 0.24, 95% confidence interval [CI] 0.08–0.70; *P* = .009). Moreover, child physical activity was not associated with demographics or neighborhood factors. Instead, children who had attention deficit hyperactivity disorder or who received occupational therapy were 4.4 (95% CI, 1.42–13.90; *P* = .01) and 4.6 (95% CI, 1.29–16.28; *P* = .02) times as likely as their cohorts to be physically active, respectively. Moreover, children whose parents were themselves physically active at least 3 hours per week were 4.2 (95% CI, 1.40–12.60; *P* = .01) times as likely to be physically active as children whose parents reported less physical activity (Table 3).

**Table 2:** Parent-Reported Barriers to Increasing Child’s Physical Activity

**Reported Barrier**	**% (n = 114)**
Parent’s lack of time	29
Inadequate community physical activity programs	21
Cannot afford the cost of exercising	25
Lacks reliable transportation	13
Cannot find program that accommodate child’s disability	33
Unsafe neighborhood	13
Child doesn’t have enough time	12
Child lacks interest/motivation	43
Child is too developmentally delayed	4
Child has too many behavioral problems	32
Child is too physically sick/frail	4

**Table 3:** Multivariate Logistic Regression Predicting Child’s Physical Activity Based on Parent-Reported Barriers and Conditions

**Predictors**	**Odds Ratio (95% CI)**	***P* Value**
Parent assessment of the child’s health^a^	2.22 (0.66–7.52)	.20
Child diagnosed with attention deficit hyperactivity disorder	4.44 (1.42–13.90)	.01
Child receiving occupational therapy	4.59 (1.29–16.28)	.02
“I (parent) do not have reliable transportation.”	0.57 (0.16–2.08)	.40
“My child does not have enough interest/motivation.”	0.24 (0.08–0.70)	.009
How much do you (parent) exercise?	4.20 (1.40–12.60)	.01
**Race/ethnicity**		
Non-Hispanic white	1 [Reference]	
Hispanic or Latino	0.46 (0.12–1.75)	.29
Black or African American	0.60 (0.16–2.29)	.46
American Indian or Alaska Native	0.44 (0.06–3.07)	.41
**Sex of parent**		
Male	0.52 (0.13–2.05)	.35

a “Very good” to “excellent” health compared with those who were ranked lower.

## Discussion

Most parents in our sample felt that their children needed to be more physically active, and a large proportion reported that their physician had made a similar recommendation in the past. However, despite numerous barriers reported by these parents, their own participation in physical activity was strongly associated with their child’s physical activity level. Lack of any significant association between the child’s physical activity level and other potential factors suggests that improving parents’ physical activity level might yield benefits. Hence, improving parental motivation and attitude toward their own physical activity might prove to be one key to increasing the physical activity level of children with special needs.

Educating parents about the types, duration, and benefits of different activities that can be incorporated into their own daily lives might play an important role in changing parental attitudes toward their own physical activity and the child’s amount of physical activity (19). The existing evidence for the efficacy of family-based interventions for healthy children may serve as a starting point in studying the efficacy of similar interventions for children with special needs and their parents.

Conceptually, improved parental attitude toward physical activity combined with increased direct involvement of parents may improve compliance with physicians’ recommendations for greater activity and break the cycle of inactivity for both parents and children (16,20). A review study by Kitzmann and Beech has shown that family-based interventions that are narrowly focused on improving the child’s obesity have had mixed results, whereas broader family-based interventions have been more successful (21). Narrowly focused family-based interventions were defined as those that aimed at improving the child’s nutrition and physical activity but that did not include “general parenting skills or family functioning” (21). Any intervention aimed at improving the long-term behavior of special needs children, therefore, might require that parents not only get involved but also that family dynamics and overall attitudes toward physical activity change. In other words, opportunities for children’s physical activity may have more impact if they actively engage parents and are accompanied by education about physical activity that can be implemented by all family members. Therefore, future research aimed at improving physical activity in children with special needs should extend beyond goals such as changing child body mass index, blood pressure, and heart rate and should also incorporate goals such as changing parental attitudes toward physical activity and increasing the activity of other family members.

Although our study did not demonstrate any correlation between most of the barriers mentioned by the parents (eg, too many behavioral problems, inadequate time, and lack of programs appropriate for children with special needs) and the amount of their child’s physical activity, these factors should still be kept in mind when designing community or school-based activity programs for children with chronic diseases. Lack of correlation might have been due to our small sample size or unmeasured variables such as family structure, child’s other commitments, or availability of other community resources.

Child’s lack of interest and lack of programs appropriate for children with special needs, for instance, were 2 barriers mentioned by parents that may be related in many ways. Children with special needs may avoid certain activities because of the difficulty of the tasks and skills needed for participation. Faigenbaum et al reported that children with atypical motor skill proficiency are more likely to engage in sedentary or “safe” activities (22). This might be especially true for children with developmental disabilities, who may lack skills needed to engage their peers or succeed competitively. For example, children with autism may not have the language or social skills needed to interact with other children in certain types of group activities. Thus, designing physical activity regimens that are developmentally appropriate and allow flexibility in developmental and physical skills may overcome this barrier.

Limitations of this study include small sample size, lack of power to detect weaker associations, and our use of a single site, which may have resulted in a nonrepresentative sample population. Reliance on parent report and subjective assessments of barriers, health, and physical activity rather than prospective observation by using objective criteria may have also introduced biases.

Our study suggests that, at least among this single group of children with special needs, child physical activity is strongly associated with parental physical activity and that commonly cited barriers specific to children with special needs may not have as direct an impact on child physical activity as parents believe. This study provides initial evidence that family-based interventions may be just as important for children with special needs as they are for healthier children.

Further studies are needed to discover the types of programs that effectively increase physical activity among children with special needs. Screening tools that help parents, educators, and physicians identify sedentary children with special needs may help prevent obesity and its adverse outcomes. Furthermore, studies of physical activity should include broader outcome measures that include parental knowledge and behavior with respect to physical activity for the entire family.
